# Hyperbaric Oxygen Ameliorates Insulin Sensitivity by Increasing GLUT4 Expression in Skeletal Muscle and Stimulating UCP1 in Brown Adipose Tissue in T2DM Mice

**DOI:** 10.3389/fendo.2020.00032

**Published:** 2020-01-31

**Authors:** Yuan Liu, Di Zhang, Junhua Yuan, Limin Song, Caishun Zhang, Qian Lin, Manwen Li, Zhi Sheng, Zhengye Ma, Fengyuan Lv, Guangkai Gao, Jing Dong

**Affiliations:** ^1^Department of Special Medicine, Basic Medical College, Qingdao University, Qingdao, China; ^2^Medical College, Qingdao University, Qingdao, China; ^3^Department of Hyperbaric Medicine, No. 971 Hospital of Chinese People's Liberation Army, Qingdao, China; ^4^Department of Physiology, Medical College, Qingdao University, Qingdao, China

**Keywords:** hyperbaric oxygen, type 2 diabetes mellitus, insulin sensitivity, glucose transporter type 4, UCP1

## Abstract

Hyperbaric oxygen (HBO) therapy is a treatment modality useful for diseases. Hypoxia could stimulate the induction of insulin resistance. Therefore, we sought to determine whether hyperbaric oxygen would ameliorate insulin sensitivity by promoting glucose transporter type 4 (GLUT4) expression in muscle and by stimulating UCP1 in brown adipose tissue (BAT) in a streptozocin (STZ)-induced type 2 diabetes mellitus (T2DM) mouse model. Male C57BL/6J mice were treated three times with low-dose of streptozocin (60 mg/kg, i.p.) and were fed with high-fat diets (HFD) to establish the T2DM model. HBO was administered daily as 100% oxygen at 2.0 atmosphere absolute (ATA) for 1 h for a week. We found that HBO significantly reduced blood glucose levels and attenuated insulin resistance in T2DM mice. HBO modulated food intake by influencing the activity of neuropeptide Y (NPY)-positive neurons in the arcuate nucleus (Arc). HBO treatment increased GLUT4 amount and level of phosphorylated Akt (p-Akt) in muscles of T2DM mice whereas this treatment stimulated the phosphorylation of AMPK in muscles of both T2DM and HFD mice. The morphological staining of BAT and the increased expression of uncoupling of protein 1 (UCP1) demonstrated the promotion of metabolism after HBO treatment. These findings suggest that HBO ameliorates insulin sensitivity of T2DM mice by stimulating the Akt signaling pathway and by promoting GLUT4 expression in muscle, and by increasing UCP1 expression in BAT.

## Introduction

Type 2 diabetes mellitus (T2DM) is the more common type of diabetes mellitus. It is characterized by hyperglycemia caused by impaired insulin secretion and peripheral insulin resistance (IR) (1); it has severe effects on human health (2). Insulin resistance is defined as inability of hormone insulin signals in tissues or cells to increase glucose uptake and utilization (3, 4).

The prevalence of diabetes is 451 million among individuals 18–99 years. Globally, about 1 in 11 adults have diabetes mellitus (90% have T2DM), and Asia is the epicenter of this global T2DM epidemic (5). The incidence of disability caused by diabetes mellitus has increased substantially (6). Metformin is the oral medication that most often initiate worldwide. There is also a range of combination therapies, including: sulphonylureas, thiazolidinediones, DPP-4 inhibitors, SGLT2 inhibitors, GLP-1 agonists, and acarbose. When oral hypoglycemic medications are not useful, insulin injections may be necessary (7). Therefore, there is an urgent need to find ways to reverse insulin resistance.

Hyperbaric oxygen (HBO) therapy is a kind of treatment modality that 100% oxygen is pressurized higher than the barometric pressure at sea level in a hyperbaric chamber (8). Hyperbaric oxygen is used to treat anemia, carbon monoxide poisoning, and ischemia (9). Hypoxia stimulate white adipocytes to produce of adipokines associated with inflammation, resulting in insulin resistance. Hypoxia also occurs in brown adipose tissue (BAT) in obese patients, compromising thermogenic activity (10, 11). The sensitivity of skeletal muscle to insulin is also reduced, as is the expression of glucose transporter type 4 (GLUT4) reducing glucose uptake and utilization and raising blood glucose levels. High levels of glycated hemoglobin (HbA1c) influence O_2_ transport to active muscles during maximal exercise (12). Studies have shown that hyperbaric oxygen enhances glucose and lipid metabolism in skeletal muscle, delaying the onset of type 2 diabetes and obesity (13). Exposure to mild hyperbaric oxygen increased skeletal muscle oxidative capacity (14). Nevertheless, it is unknown as to whether HBO improves insulin sensitivity and relative mechanism.

Therefore, we determined whether HBO increases insulin sensitivity, promoting GLUT4 expression in muscle as well as energy metabolism in BAT in a mouse model of T2DM. This might provide evidence of HBO as a possible treatment for T2DM.

## Materials and Methods

### Animals and Treatments

Male C57BL/6J mice (5 weeks of age) weighing >15 g upon arrival were purchased from Qingdao Institute of Drug Control. They had free accessed to food and water in a 12 h light/dark cycle and were housed in standard housing conditions (22–24°C and 55 ± 20% humidity, illumination from 7:00 to 19:00). The protocols used for handling the mice were approved by Qingdao University Animal Care and Use Committee and were in accordance with the National Institutes of Health guidelines.

**Figure 1:**
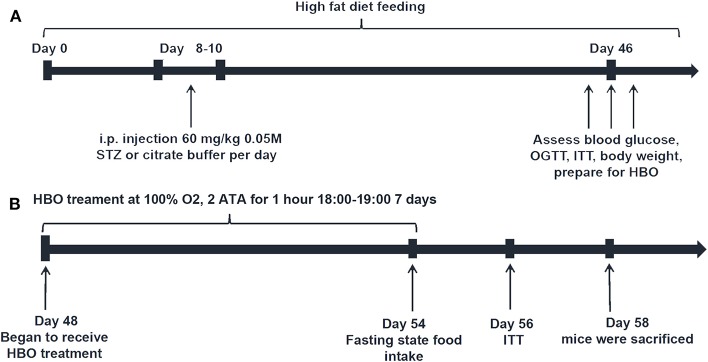
Schedule diagram. **(A)** Type 2 diabetic model building. **(B)** HBO treatment protocol.

The mice were randomly divided into four groups: high fat diet group (HFD), high fat diet with 1-week HBO treatment group (HFD+HBO), type 2 diabetes mice group (T2DM) and type 2 diabetes mice with 1-week HBO treatment group (T2DM+HBO). To establish the type 2 diabetes mouse model (Figure 1A), mice were fed a high-fat diet (59% basic mice feed, 20% sugar, 18% lard, and 3% egg yolk). The first day of high fat diet feeding was designated day 0. Then low-dose streptozocin (STZ, 60 mg/kg, i.p., S0130, Sigma-Aldrich) was injected into mice at 10:00 after 15-h fasting on days 8–10 after 1-week high-fat diet feeding. Age-matched HFD mice were injected with citrate buffer alone (15). Low dose STZ injection with high-calorie diet caused peripheral tissues to become insensitive to insulin, thereby establishing the T2DM mice model. STZ was dissolved in citric acid buffer (0.05 M, pH 4.5) consisting of citric acid (5.22 g/L) and sodium citrate (7.39 g/L). Only mice with fasting blood glucose levels above 12 mmol/L (16) were considered successful type 2 diabetic models.

From day 48, both HFD+HBO and T2DM+HBO groups began to receive 1-h HBO treatment daily from 18:00 to 19:00 for 1 week, while HFD and T2DM group were also placed in the same chamber with normobaric oxygen condition (NBO) (Figure 1B). HBO treatment was administered in an animal hyperbaric oxygen chamber (Moon Environmental Technology Co., Ltd), operating at high pressure (2 ATA) with 100% oxygen. Animals were sacrificed 2-h after the final HBO treatment in the morning on day 58, with food withdrawal 12-h before sacrifice. Blood samples were obtained from the orbital sinus and stored at −20°C while other samples were stored at −80°C until use.

### Food Intake and Body Weight

After 1-week of HBO treatment, mice were treated with the 7th HBO for 1 h following 11-h food deprivation (7:00–18:00). Then food intake after refeeding was measured using an electronic precision scales (TE412-L; Sartorius, Göttingen, Germany). All the remaining food including the spillage was weighed every hour as previously described Wang et al. (17). Body weights were measured using an electric balance (Mettler Toledo, PL1501-S, Shanghai, China).

### Blood Glucose, Oral Glucose Tolerance Test (OGTT), Intraperitoneal Insulin Tolerance Test (ITT), and HOMA-IR

Blood glucose was measured using a tail vein prick and a glucometer (B, Braun, Meisungen AG, Germany). OGTT was performed following the final type 2 diabetic model building period (Figure 1B). The mice were fasted for 14-h before the OGTT and then were treated with glucose (3 g/kg body weight) orally. Blood glucose concentration was measured at 0, 15, 30, 60, 90, 120 min by tail vain blood after gavage using a glucometer. After building the diabetic model mice and the 1-week HBO treatment, ITT was performed to assess the insulin resistance following 4-h fasting. Mice received 0.75 unit/kg of regular insulin (NovoMix® 30 Penfill®, Novo Nordisk®) by i.p. injection and we measured blood glucose levels at 0, 15, 30, 45, 60, and 90 min after insulin injection. Insulin (CEA448Mu, Wuhan USCN Business Co., Ltd) and leptin (#JL11317, Shanghai Jianglai industrial Limited By Share Ltd) levels of blood samples were determined using a mouse ultrasensitive ELISA Kit. The homeostasis model assessment index (HOMA-IR) was calculated as follows: fasting plasma insulin (mU/ml) × fasting plasma glucose (mmol/L)/22.5 (18).

### Histological Assessment

#### Immunofluorescence

Brains were fixed in 4% paraformaldehyde, and then dehydrated in 30% sucrose. Brains were frozen and sectioned at 15-μm thickness using a cryostat microtome (CM1950, LEICA) and were placed on glass microscope slides. After microwave repair for the antigens for 10 min, blocking solution (1% BSA+0.1% Tween-20 in 0.01M PBS) was added. We used primary sheep antibodies for neuropeptide Y (NPY, 1:100, Abcam) in universal antibody diluent (WB100D, NCM Biotech) incubated at 4°C overnight. Then sections were incubated slides with donkey anti-sheep secondary antibody (NL101, 1:200; R&D Systems, Minneapolis, MN, United States) for 2 h and coverslips were mounted with a drop of 50% glycerol. An Olympus laser scanning confocal microscope (FV500, Carl Zeiss, Oberkochen, Germany) was used to measure luminescence. The intensity of immunofluorescence was quantified using Image J (version 1.8.0).

For the evaluation of GLUT4 levels, some muscle biopsy samples were embedded in Tissue Tek OCT compound (Sakura) and immediately frozen for storage at −80°C. The remaining samples were snap frozen in liquid nitrogen to be used for western blot analysis and stored at −80°C. For immunofluorescence staining frozen muscle biopsy samples were cryosectioned using a microtome within a cryostat to a thickness of 5-μm and were placed on glass microscope slides. The following immunostaining protocols were as described above. Blocking buffer: 1% BSA in 0.01M PBS. Primary antibody: Rabbit Anti-Glucose Transporter GLUT4 polyclonal antibody (1:1,000, ab654, Abcam), incubated overnight at 4°C; Secondary antibody: Fluorescein-Conjugated AffiniPure Goat Anti-Rabbit IgG (H+L) (1:200, ZSGB-BIO), incubated for 2 h at room temperature.

#### Immunohistochemistry

Pancreatic tissues were rinsed after fixing in 4% formaldehyde for 24 h and then in 70, 85, 95, 95, 100, 100% ethanol for 30 min each. Then tissue was transferred into dimethylbenzene for 4 min, following immersion in soft wax, hard wax, and mixed waxes for 60 min, respectively. cut into 5-μm sections and placed in 100, 100, 90, 80, 70% ethanol for 5 min, respectively. This was followed by dimethylbenzene dewaxing for 10 min. After washing in 0.01 M PBS, the following immunostaining protocols as described above: blocking buffer: 1% BSA in 0.01M PBS. Primary antibody: Rabbit anti-insulin antibody (1:200, #4590, Cell Signaling Technology), incubated overnight at 4°C; HRP (horse radish peroxidase) secondary antibody (PV-6001; Zsbio, Tianjin, China) for 20 min at 37°C. They were then stained using a DAB Kit (ZLI-9018; Zsbio). Morphology was assessed using a light microscope (CX31; Olympus, Tokyo, Japan). β-cell mass area ratio was determined analyzed using Image-Pro Plus software.

#### Hematoxylin-Eosin (HE) Staining

Some BATs were fixed in 4% formaldehyde for 24 h. After dehydration with various concentrations of ethanol and clarifying with xylene, tissues were embedded in mixed paraffin prior to sectioning. BATs were cut into 5-μm sections using a paraffin slicing machine (RM2016; Leica, Wetzlar, Germany), respectively. Sections were stained using a HE Staining Kit (G1120; Solarbio, Beijing, China). Cell numbers were counted using Image J (version 1.8.0).

### Western Blot Analysis

Muscle strips and BATs were homogenized in ice-cold standard RIPA buffer (Beyotime, P0013B, China), followed by centrifugation at 4°C for 15 min at 12,000 *g*. BCA Protein Assay Kit (P0012; Beyotime, Shanghai, China) and a microplate reader (M5; MD-SpectraMax, Molecular Devices, San Jose, CA, United States) were used to determine protein concentrations. Equal amounts (35 μg/10 μl) of protein were subjected to SDS-PAGE, followed by transfer to polyvinylidene fluoride membranes (PVDF, IPVH00010: Millipore, Burlington, MA, US), that were activated with methanol. After blocking with 5% skimmed milk powder in TBST (pH = 7.6–7.9) for 2 h at room temperature, we used primary antibodies in 3% fetal bovine serum (9048-46-8, Solarbio) incubated at 4°C overnight (UCP1: rabbit IgG, 1:2,000, ab10983, Abcam, US; GLUT4: rabbit IgG, 1:2,000, Abcam, ab654, US; Akt: rabbit IgG, 1:2,000, Sigma, SAB4500797, Germany; phosphorylated-Akt (p-Akt): rabbit IgG, 1:2,000, Cell Signaling Technology, PSER473, #4060, USA; Phosphorylated- AMPKα (Thr172, #2535) and AMPKα (#2532S): rabbit IgG, 1:2,000, Cell Signaling Technology; β-actin: rabbit IgG, 1:2,000, Cell Signaling Technology, D6A8, #8457, USA; GAPDH: rabbit IgG, 1:1,000, Cell Signaling Technology, #5174, USA). The secondary antibody was goat anti-rabbit IgG H&L (HRP) (1:4,000, ZB-2306 ZSGB-BIO, China) incubated at room temperature for 1 h. The protein bands were developed by using Immobilon Western Chemiluminescent Substrate (Millipore, WBKLS0100).

### Statistical Analysis

All data were presented as means ± standard error of the means (SEMs). Comparisons between two groups were made using the Student's *t*-test. two-way ANOVA followed by a *post hoc* least significance difference test was used for comparisons among three groups. The least significance difference test was used as *post hoc* test. *P* < 0.05 denoted statistical significance. GraphPad Prism and SPSS (Statistical Product and Service Solutions) were used to create graphs and perform statistical analyses.

## Results

### T2DM Mouse Model

**Figure 2:**
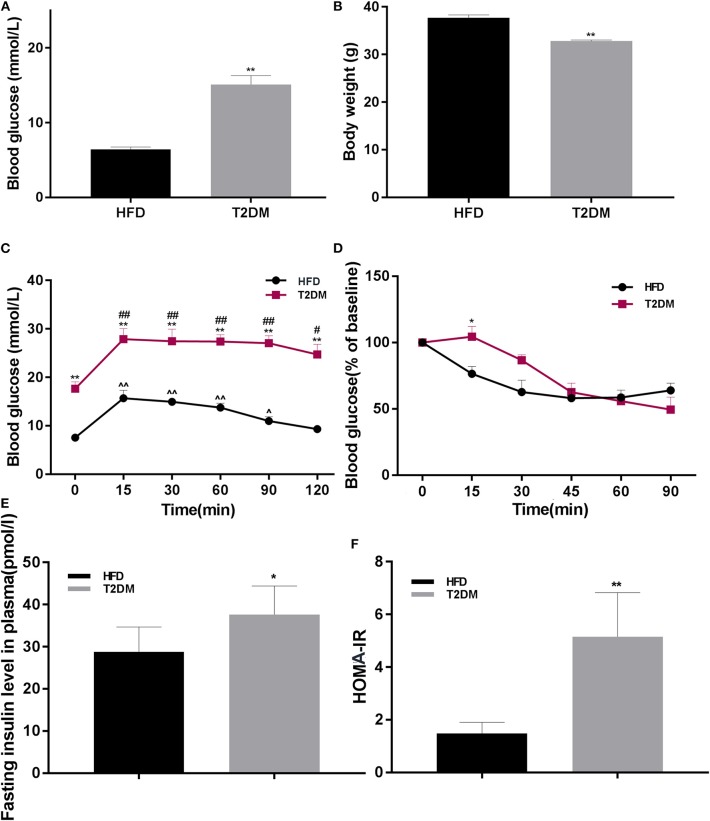
The differences between HFD and T2DM mice in terms of blood glucose levels, body weight, OGTT, ITT, insulin concentration, and HOMA-IR. **(A)** Blood glucose in HFD and T2DM mice under 12-h fasting conditions after successful induction of diabetes. **(B)** Changes in body weight over 46 days. **(C)** Oral glucose tolerance tests (OGTT) in mice fed with high-fat diet. **(D)** Insulin tolerance tests (ITT) in HFD and T2DM mice. **(E)** Insulin concentration and **(F)** HOMA-IR values between HFD and T2DM mice. Data are presented as means ± SEM (HFD: *n* = 13; T2DM: *n* = 15). *P < 0.05, **P < 0.01 (compared with HFD group), ^#^*P* < 0.05, ^##^*p* < 0.01 (compared with 0 min blood glucose in T2DM group), and ^∧^*P* < 0.05, ^∧∧^*P* < 0.01 (compared with 0 min blood glucose in HFD group).

The rate of successful type 2 diabetic model creation was about 70%. Blood glucose in T2DM mice was higher than only that of the HFD fed group (*P* < 0.01; Figure 2A). We found significantly lower body weights in T2DM mice (*P* < 0.01; Figure 2B). To further confirm effects on glucose metabolism and insulin sensitivity, we performed OGTT and ITT, suggested impaired glucose tolerance (Figure 2C) and insulin resistance (*P* < 0.05; Figure 2D). Consistently, T2DM mice also showed high insulin levels (*P* < 0.05; Figure 2E) and higher HOMA-IR values (*P* < 0.01; Figure 2F).

### The Change of Fasting Blood Glucose and Body Weight After Exposure to HBO or NBO

**Figure 3:**
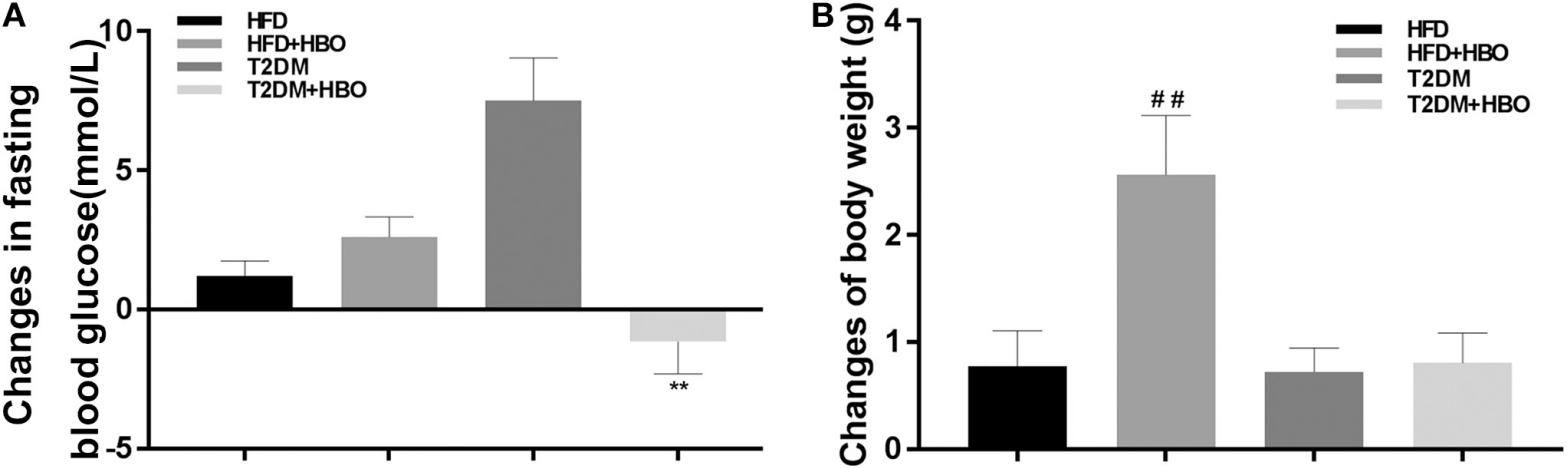
Changes in blood glucose, body weight gained after 7-day HBO or NBO treatment. **(A)** The blood glucose changes pre- and post HBO treatment. **(B)** HBO-induced body weight gained after 7-day HBO treatment. Data are presented as means ± SEM (HFD: *n* = 6; HFD+HBO: *n* = 7; T2DM: *n* = 7; T2DM+HBO: *n* = 8). **P < 0.01 (compared with T2DM group), ^##^*P* < 0.05 (compared with HFD group).

HBO significantly reduced fasting blood glucose (*P* < 0.01; Figure 3A) in T2DM mice. HBO also gave rise to significantly higher body weight gain in the HFD group (*P* < 0.01); Nevertheless, no significant difference was observed in T2DM mice (*P* > 0.05; Figure 3B).

### HBO Increased Cumulative Food Intake and NPY-Positive Neurons Expression in the Arcuate Nucleus (Arc) in T2DM

**Figure 4:**
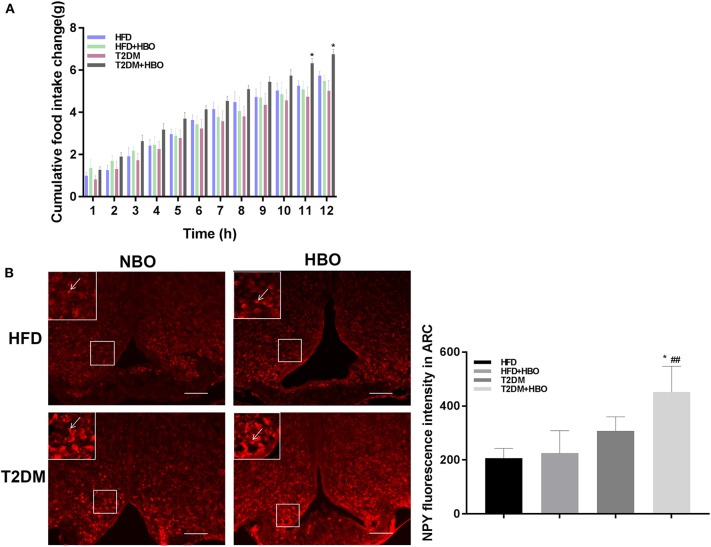
HBO influenced feeding responses and central NPY-positive neurons expression in the Arc. **(A)** After 12-h food deprivation, the HBO increased nocturnal cumulative food intake in the last 2 h of monitoring in T2DM. **(B)** NPY immunoreactive cell detection in Arc (200×, picture in upper-lift corner is 400×). Numbers of NPY-positive neurons were counted. Data are presented as means ± SEM(HFD: *n* = 6; HFD+HBO: *n* = 7; T2DM: *n* = 7; T2DM+HBO: *n* = 8). Normobaric oxygen (NBO). Scale bar = 100 μm. *P < 0.05 (compared with T2DM group), ^##^*P* < 0.01 (compared with HFD+HBO group).

It is interesting to note that cumulative food intake in T2DM+HBO showed a significantly increasing tendency observed in the last 2 h (*P* < 0.05), while no significant alteration in cumulative food intake was found between the HFD and HFD+HBO groups (*P* > 0.05; Figure 4A). To further study whether HBO modulated the food intake by influencing the activity of neurons in the hypothalamus, NPY-positive neurons in Arc were counted after 7 days of HBO treatment. Compared with the untreated T2DM group, NPY-activated neurons in the T2DM+HBO group were increased in the Arc in each section (*P* < 0.05; Figure 4B); however, there was no significant difference between HFD groups. Leptin are linked to appetite regulation so the level of leptin in plasma was measured, but there was no significant differences in leptin level between the groups of mice (data not shown).

### HBO Improved Insulin Sensitivity and HOMA-IR in T2DM

**Figure 5:**
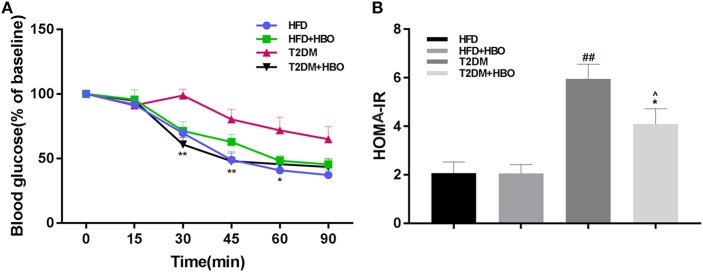
Effects of HBO on insulin sensitivity and HOMA-IR coefficient. **(A)** Insulin tolerance tests (ITT) in HFD and T2DM mice after HBO treatment. **(B)** The insulin-resistance coefficient. HOMA IR = fasting plasma insulin (mU/ml) × fasting plasma glucose (mmol/L)/22.5. Data are presented as means ± SEM (HFD: *n* = 6; HFD+HBO: *n* = 7; T2DM: *n* = 7; T2DM+HBO: *n* = 8). *P < 0.05, **P < 0.01 (compared with T2DM group), ^∧^*P* < 0.05 (compared with HFD+HBO group), ^##^*P* < 0.01 (compared with HFD group).

To assess insulin resistance, ITT was performed. This showed that HBO significantly improved insulin sensitivity (*P* < 0.01; Figure 5A) from the 30th min compared with untreated HBO in T2DM mice. As expected, the insulin resistance coefficient was obviously higher in T2DM mice than in HFD mice (*P* < 0.01), while HBO treatment lower the coefficient significantly (*P* < 0.05; Figure 5B). There were no differences between HFD mice with and without HBO.

### Pancreatic β Cell Mass Were Increased in T2DM After HBO Treatment

**Figure 6:**
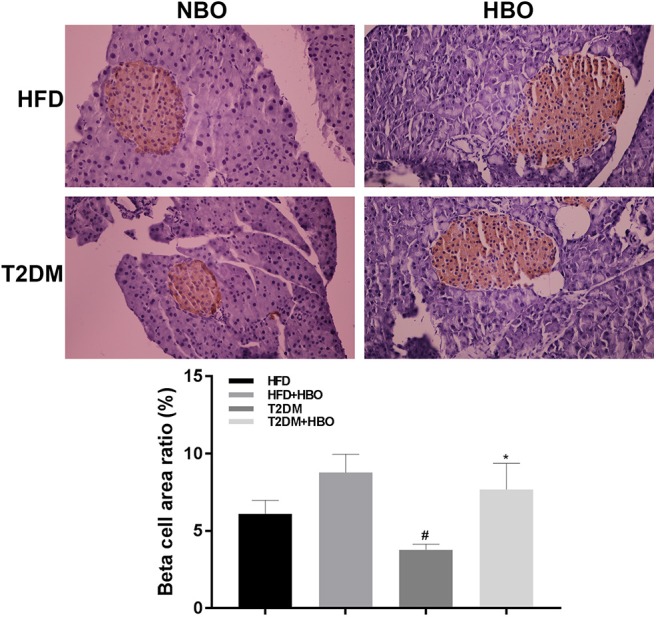
Effect of HBO on β cell mass. Pancreas were cut into 5-μm sections stained with an anti-insulin antibody by immunohistochemical staining (400×) and area ratio of β cell mass were counted. Data are presented as means ± SEM (HFD: *n* = 6; HFD+HBO: *n* = 7; T2DM: *n* = 7; T2DM+HBO: *n* = 8). *P < 0.05 (compared with T2DM group), ^#^*P* < 0.05 (compared with HFD group).

After injection of STZ to induce T2DM model, β-cell mass reduced in pancreatic tissue. However, HBO treatment significantly increased β-cell mass about 2-fold without HBO in T2DM (*P* < 0.05) by promoting β-cells proliferation. The same tendency was also seen in the HFD group followed HBO exposure, although without statistical significance (Figure 6).

### HBO Treatment of T2DM Mice Increases GLUT4 Expression in Myocyte, and Akt and AMPK Phosphorylation in Skeletal Muscle

**Figure 7:**
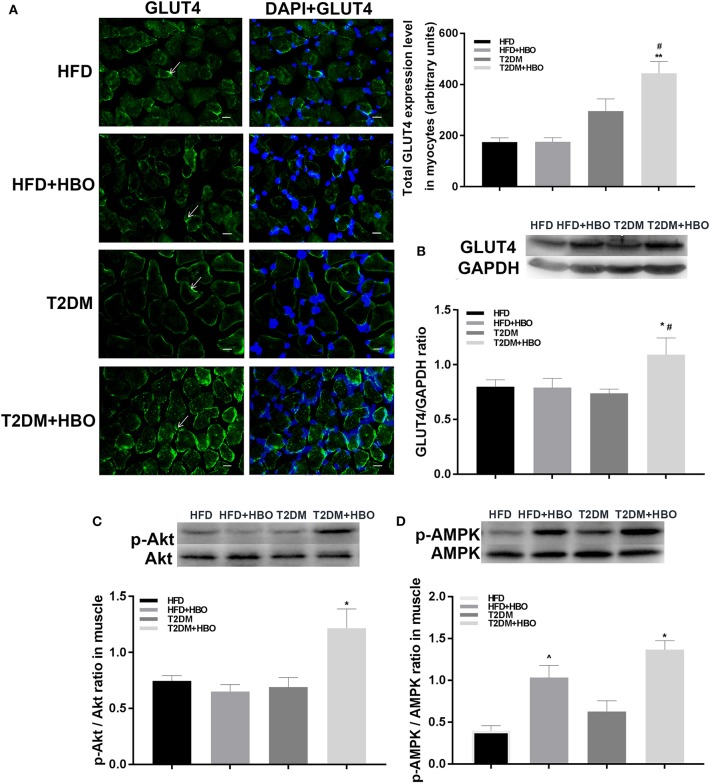
Effect of HBO on levels of GLUT4 in skeletal muscle, Akt, and AMPK phosphorylation. **(A)** GLUT4 expression levels detected by immunofluorescence in skeletal muscle (400×) and measured intensity per arbitrary units. Arrows point to GLUT4 immunoreactive cells. Blue is DAPI (HFD: *n* = 3; HFD+HBO: *n* = 3; T2DM: *n* = 4; T2DM+HBO: *n* = 4). **(B)** GLUT4 levels in skeletal muscle measuring by western blot. **(C)** Western blot of p-Akt/Akt level in skeletal muscle (*n* = 3 per group). **(D)** Western blot of p-AMPK/AMPK level in skeletal muscle (*n* = 3 per group). Scale bar = 200 μm. Data are presented as means ± SEM. *P < 0.05, **P < 0.01 (compared with T2DM group), ^#^*P* < 0.05 (compared with HFD+HBO group), ^∧^*P* < 0.05 (compared with HFD group).

Next we investigated the possible molecular mechanisms of glucose-regulating effects of HBO. First, we measured GLUT4 expression levels using immunofluorescence. HBO elevated GLUT4 expression in T2DM (*P* < 0.05), and this effect was not seen in HFD mice (Figure 7A). Next, we measured expression levels of GLUT4 by western blot after 1-week HBO treatment in skeletal muscle. We found significantly greater expression levels of GLUT4 in skeletal muscle in the T2DM+HBO group than in the T2DM group (*P* < 0.05); however, the result of protein levels was inconclusive in HFD mice (Figure 7B). We then examined phosphorylation levels of Akt as markers of the insulin signaling pathway and AMPK that regulates energy metabolism in skeletal muscle. HBO increased phosphorylation levels of AMPK (*P* < 0.05) and Akt (*P* < 0.01) in skeletal muscle in the T2DM+HBO group; however HBO increased phosphorylation levels of AMPK rather than Akt in HFD+HBO group (Figures 7C,D).

### BAT Cell Density and UCP1 Expression Increased in T2DM After Exposure to HBO

**Figure 8:**
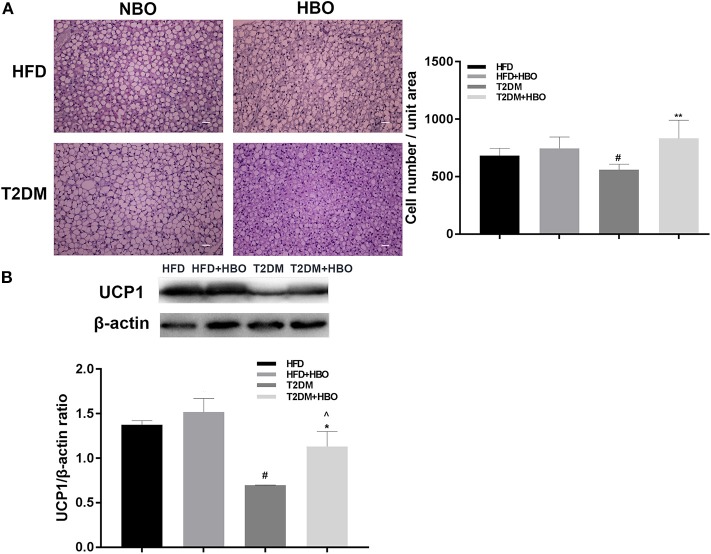
**(A)** Morphology of BAT and cell number counting. BAT morphology and UCP1 protein analysis after HBO. Partial BATs were cut into 5-μm sections stained using hematoxylin-eosin staining (400×) and numbers of cell from the same unit areas were counted (HFD: *n* = 3; HFD+HBO: *n* = 4; T2DM: *n* = 3; T2DM+HBO: *n* = 4). **(B)** UCP1 protein expression levels in BAT were measured (HFD: *n* = 3; HFD+HBO: *n* = 3; T2DM: *n* = 4; T2DM+HBO: *n* = 4). Data are presented as means ± SEM. Normal baric oxygen (NBO). Scale bar = 200 μm. *P < 0.05, **P < 0.01 (compared with T2DM group), ^#^*P* < 0.05 (compared with HFD group), ^∧^*P* < 0.05 (compared with HFD+HBO group).

We measured the morphology of BAT and UCP1 expression to further determine whether HBO was related to the change in BAT in addition to energy metabolism. After 7 days of HBO, there were significantly increased numbers of cells (*P* < 0.01; Figure 8A) and increased the protein expression levels of UCP1 (*P* < 0.05; Figure 8B) in BAT than in those untreated in T2DM. Moreover, UCP1 expression levels in BAT adipose pads were significantly greater in HFD though cell numbers while there was only an increased tendency in the HFD+HBO group.

## Discussion

We first observed that HBO can increase insulin sensitivity by promoting GLUT4 expression in muscle as well as energy metabolism in BAT in a mouse model of T2DM. This might provide evidence of HBO as a possible treatment for T2DM. The characteristic features of T2DM and obesity are metabolic defects in which the organism is unable to adequately regulate energy homeostasis. A change in body weight can result from altering the balance between food intake and energy expenditure. Bodily functions and activities require energy from food intake that is influenced by conditions, including starvation, stress and others (19). The central nervous system regulates food intake by the key primary neurons in the Arc, anorexigenic neurons that express proopiomelanocortin inhibiting feeding and orexigenic neurons that express NPY to stimulate food intake (20). In the present study, we found that cumulative food intake increased in T2DM after HBO treatment as well as NPY-positive neurons expressed in Arc while body weight showed no change, suggesting that HBO treatment improved metabolism in T2DM mice. By contrast, HFD mice showed a decreasing tendency in food intake with body weight increasing after exposure to HBO. Oxidation of fatty acids is closely related to regulation of food intake (21). Leptin levels are usually increased in the insulin resistant models and are linked to appetite regulation (22). Therefore, one possible explanation is that HBO exerts its anorexic role in HFD-mice by regulating lipid metabolism. Though there is no significant change of leptin in plasma, this does not rule out a contribution from other adipokines or gut peptides such as GLP-1 or GIP.

Skeletal muscle plays an important role in glucose regulation and is the principal site of glucose metabolism. Glucose uptake into skeletal muscle is principally regulated by GLUT4 (23, 24). GLUT4 is the only transporter responsible for facilitating glucose transport into the cells in response to insulin and the modulation of IRS1-Akt-GLUT4 signaling pathway. Therefore, it thought to be an important regulator of glucose homeostasis for the organism as a whole (25, 26). Abnormality of proteins including Akt and GLUT4 in the phosphatidylinositol 3-kinase signaling pathway cause T2DM (27, 28). Our results suggest that treatment with hyperbaric oxygen improves insulin sensitivity, further activating Akt protein phosphorylation to promote GLUT4 expression in T2DM, resulting in decreased blood glucose levels. Stimulation of glucose utilization and fatty-acid oxidation by adiponectin occurs through activation of AMPK which is an insulin-independent pathway that regulates energy metabolism (29), though Hypoxia-inducible factor-1α (HIF-1α) regulates glycolysis in cancer cells by inactivation of AMPK (30). In our study, HBO increased AMPK activity in skeletal muscle. Previous study found that exposure to mild hyperbaric oxygen increased skeletal muscle oxidative capacity (14). So HBO may promote oxidative utilization of glucose in skeletal muscle in T2DM and fatty acid oxidation in HFD. Our findings are consistent with those of previous studies showing that HBO improved fasting blood glucose levels in diabetes patients (31, 32) and ameliorated glucose tolerance (33). By contrast, for HFD mice, HBO may mainly influence other tissues such as adipose tissue metabolism because no changes of GLUT4 in skeletal muscle were found after HBO exposure. We hypothesized that the response of HBO on regulating glucose metabolism mediated by Akt and AMPK in muscle occurred in situations of STZ-induced insulin resistance. Further study is needed to explore this issue. We also elucidated a possible pathway by which HBO decreases blood glucose via GLUT4 for treatment of T2DM.

Central NPY-positive neurons are involved in the modulation of feeding and body weight (34). The arcuate nucleus NPY regulates various physiological functions, including BAT thermogenesis (35). BAT contains abundant mitochondria and shows high expression of UCP1, which increase heat energy dissipation (36). Previous studies found that the Arc-specific NPY signaling pathway decreased sympathetically innervated BAT thermogenesis by down-regulating of UCP1 expression and BAT temperature (37). By contrast, specific knockout of NPY in the DMH led to increased expression of UCP1 in BAT, and enhanced browning of inguinal white adipocytes (38, 39). This suggests that NPY and UCP1 exert opposite effects, but in our study, they are both increased after exposure to HBO. For the possible reason is that there may be another mechanism influencing UCP1 expression. Our UCP1 expression results were consistent in the HFD group. Nevertheless, we found that HBO significantly increased cell density in BAT and up-regulated UCP1 expression in BAT, though the cumulative food intake and NPY- positive neurons increased in the T2DM group. Despite the fact that we did not measure BAT temperatures, these results are consistent with our working hypothesis that HBO may be useful for increasing energy expenditure and maintaining body weight.

The major mechanism of HBO is increase in partial pressure of gases in the tissues, with concomitant decrease in volume of gas-filled spaces, including the bubbles (40). Elevation in partial pressure of oxygen (PO_2_) in tissue may mediate the therapeutic benefits of HBO (41). Increases in the glucose and lipid metabolic capacities of skeletal muscle were also found after long-term endurance exercise that was associated with increased PO_2_ (42–44). Therefore, it is reasonable to regard HBO as a therapy for T2DM because HBO has an effect on influencing the glucose tolerance and insulin sensitivity. Nevertheless, the mechanism of PO_2_ elevation in tissue improving insulin sensitivity remains unknown, apart from the fact that Akt phosphorylation is increased. This hypothesis requires verification through further studies.

In conclusion, HBO improves insulin sensitivity by stimulating the Akt and AMPK signaling pathway, promoting GLUT4 expression in skeletal muscle and UCP1 in BAT in T2DM mice.

## Data Availability Statement

The analyzed data sets generated during the study are available from the corresponding author on reasonable request.

## Ethics Statement

The animal study was reviewed and approved by Animal Ethics Committee of Qingdao University.

## Author Contributions

YL, DZ, and JD designed the experiments. YL, LS, QL, ML, ZS, and ZM performed the experiments. YL, JY, LS, and FL analyzed the data. CZ, QL, FL, and GG interpreted results of experiments and prepared figures. YL, JY, LS, QL, and ML drafted the manuscript. DZ and JD revised the manuscript. All authors reviewed the manuscript and agreed to be accountable for the content of the work.

### Conflict of Interest

The authors declare that the research was conducted in the absence of any commercial or financial relationships that could be construed as a potential conflict of interest.
